# Identification of key genes associated with acute myocardial infarction using WGCNA and two-sample mendelian randomization study

**DOI:** 10.1371/journal.pone.0305532

**Published:** 2024-07-18

**Authors:** Xiaohe Yang, Yingtao Huang, Dadong Tang, Liangming Yue

**Affiliations:** 1 Department of Cardiology, Guangyuan Hospital of Traditional Chinese Medicine, Guangyuan, China; 2 Department of Orthopedics, Liaoning University of Traditional Chinese Medicine, Shenyang, China; 3 School of Clinical College of Medicine, Chengdu University of Traditional Chinese Medicine, Chengdu, China; BSMMU: Bangabandhu Sheikh Mujib Medical University, BANGLADESH

## Abstract

**Objective:**

Acute myocardial infarction (AMI) is a severe condition with high morbidity and mortality rates. This study aimed to identify hub genes potentially associated with AMI and assess their clinical utility in predicting AMI occurrence.

**Methods:**

Gene microarray data were obtained from the Gene Expression Omnibus (GEO) database. Differential expression analysis and weighted gene co-expression network analysis (WGCNA) were conducted on samples from patients with AMI and control samples to identify modules significantly associated with AMI. GO and KEGG analyses were applied to investigate the potential functions of these hub genes. Lastly, the mendelian randomization (MR) method was applied to analyze the causal relationship between the hub gene TNF and AMI.

**Results:**

285 differentially expressed genes (DEGs) were identified through WCGNA and were clustered into 6 modules. The yellow module appeared most relevant to AMI. Further exploration through GO and KEGG pathway enrichment showed that key hub genes in the yellow module were linked to positive regulation of cytokine production, cytokine receptor binding, NF—kappa B signaling pathway, IL−17 signaling pathway, and TNF signaling pathway. The top 10 genes identified through Cytoscape software analysis were IL1B, TNF, TLR4, TLR2, FCGR3B, MMP9, CXCL8, TLR8, ICAM1, and JUK. Utilizing inverse variance weighting (IVW) analysis, we discovered a significant association between TNF and AMI risk, with an OR of 0.946 (95% CI = 0.911–0.984, p = 0.005).

**Conclusions:**

The result of this study indicated that TNF, TLR2, TLR4, IL1B and FCGR3B may be potential biodiagnostic markers for AMI. TNF can inhibit inflammatory and oxidative stress responses in AMI, exerting a protective role in the heart.

## 1. Introduction

Acute myocardial infarction (AMI) refers to acute myocardial injury caused by a sudden reduction in blood flow in a specific area of the heart. It stands as the most serious acute condition among cardiovascular diseases, with high morbidity and mortality rates [1]. Diagnosis of AMI includes clinical symptoms, electrocardiogram, and sensitivity analysis of serum biomarkers. The use of biomarkers, such as cardiac troponin, has significantly enhanced clinicians’ ability to diagnose AMI. However, in certain patients with cardiomyopathy, the normal renewal of myocardial cells, the release of proteolytic troponin degradation products, and increased cell membrane permeability can also result in elevated levels of these biomarkers [2]. Over the past 30 years, with the clinical application of percutaneous coronary intervention (PCI), significant progress has been made in the treatment of AMI, leading to a decline in its mortality rate. Nevertheless, the number of people developing heart failure after surgery is increasing, seriously affecting the patient’s quality of life [3, 4]. Early, prompt, accurate diagnosis and efficient evidence-based treatment measures are crucial in enhancing the survival rate of AMI patients. Hence, it is imperative to explore more AMI-related specific biomarkers.

With the rapid advancements in bioinformatics and microarray analysis, an increasing number of studies in the cardiovascular field are employing combined bioinformatics methods. In previous studies, microarray expression profiling was performed on peripheral blood samples from AMI patients to identify hub genes associated with AMI [5, 6]. Weighted gene co-expression network analysis (WGCNA) is a systems biology method commonly utilized to analyze network relationships and molecular mechanisms. It has been extensively utilized in screening therapeutic targets or biomarkers [7]. WGCNA constructs a gene expression network by clustering highly correlated genes into co-expression modules, enabling the exploration of the association between sample characteristics and gene modules [8].

Mendelian Randomization (MR) is a method employed in genetic epidemiology to deduce the causal connection between exposure factors and outcomes by leveraging genetic variation as instrumental variables (IVs) [9]. This method relies on data from published genome-wide association studies (GWAS) and utilizes single-nucleotide polymorphisms (SNPs) as instrumental variables. MR can eliminate biases caused by confounding factors and reverse causality, making the results more reliable.

In this study, we analyzed data from both the AMI and control groups to identify genes that were differentially expressed. Subsequently, we employed WGCNA to identify modules most relevant to AMI and visualize the genes that best represent AMI. Through this analysis, we identified five hub genes, namely TNF, TLR2, TLR4, IL1B, and FCGR3B. Finally, we selected the hub gene TNF, most relevant to AMI, to conduct MR studies with AMI.

## 2. Methods

### 2.1. Data source

The GEO (https://www.ncbi.nlm.nih.gov/geo/) is an international public repository housing diverse datasets, including microarrays, next-generation sequencing, and other high-throughput functional genomic data [10]. For this study, we retrieved microarray data from GEO using the keyword ’myocardial infarction’. The dataset GSE66360 was downloaded from GEO. This dataset comprises circulating endothelial cell samples collected from 49 AMI patients and 50 controls.

### 2.2 Identification of differentially expressed genes

We used R statistical software (version 4.3.1) to normalize the GSE66360 dataset. After preprocessing, differential analysis was performed using the "limma" package in R to identify DEGs between the AMI and control groups. The threshold was set at P<0.05 and |log2 fold change (FC)| ≥1 to screen DEGs. Expression heatmaps and volcano plots for DEGs were created using the "pheatmap" and "ggplot2" packages in R software.

### 2.3 Weighted gene co-expression network analysis

WGCNA is a method to study co-expression patterns and biological network structures in gene expression data [11]. The analysis involves several key steps. First, cluster analysis is performed on the samples to identify and remove those with abnormal clustering. Second, the one-step network construction function establishes a scale-free co-expressed gene network. The R function "pickSoftThreshold" is used to calculate the soft threshold (β), enhancing co-expression similarity for adjacency calculation. Third, the adjacency matrix is transformed into a topological overlap matrix (TOM) and calculating the corresponding dissimilarity (1-TOM). Hierarchical clustering and dynamic tree-cutting functions are utilized to detect modules within the network. Fourth, gene significance (GS) and module membership (MM) are calculated, enabling the identification of modules associated with clinical attributes. Lastly, visualizing feature gene networks.

### 2.4 Enrichment analysis

The intersecting genes were identified as potential hub genes associated with the mechanism of AMI. Gene Ontology (GO) functional analysis and Kyoto Encyclopedia of Genes and Genomes (KEGG) pathway enrichment for these candidate genes were performed using the "clusterProfiler" package in R [12]. GO functional enrichment analysis covered biological process (BP), cellular component (CC), and molecular function (MF) categories. The significance threshold for enrichment was set at p<0.05.

### 2.5 Protein interaction network construction and hub gene identification

We utilized the STRING database (http://string-db.org) to build a network for protein-protein interactions (PPI) of DEGs. Subsequently, the PPI network was visualized using Cytoscape software. Significant interacting genes were identified through the CytoHubba plug-in of Cytoscape, and 10 core genes were chosen for further analysis.

### 2.6 Nomogram construction

The "rms" package was utilized to construct a nomogram for predicting the risk of AMI based on these signature genes. In the nomogram, points on the first line represent individual scores corresponding to each gene under different values. The total points on the last line indicate the sum of individual scores for all genes. Subsequently, the incidence of AMI was estimated based on the total score. ROC curve was utilized to assess the specificity of hub genes for AMI diagnosis. The Area under the ROC Curve (AUC) was used to assess the accuracy of these hub genes in diagnosing AMI.

### 2.7 Immune cell infiltration analysis

To evaluate the abundance of infiltrating immune cells in gene expression profiles associated with AMI, we utilized the CIBERSORT algorithm (https://cibersortx.stanford.edu/). This algorithm calculated the relative proportions of 22 distinct immune cell subtypes (LM22) within the samples.

### 2.8 Mendelian randomization

In the two-sample MR study, data from the GWAS (https://gwas.mrcieu.ac.uk/) were downloaded to investigate the causal relationship between TNF and AMI. SNPs were used as IVs. Due to the limited number of SNPs meeting genome-wide significance, a more lenient threshold (p<5×10^−6^) was applied to screen SNPs. Ultimately, the most pertinent gene, TNF, and the disease AMI were selected for MR analysis. MR analysis mainly uses the inverse variance weighting (IVW) method of random effects and fixed effects, and the“TwoSampleMR” software package in R is used for statistical analysis. Furthermore, we conducted tests of pleiotropy of the effect estimates.

## 3. Results

### 3.1 Identification of DEGs

**Fig 1:**
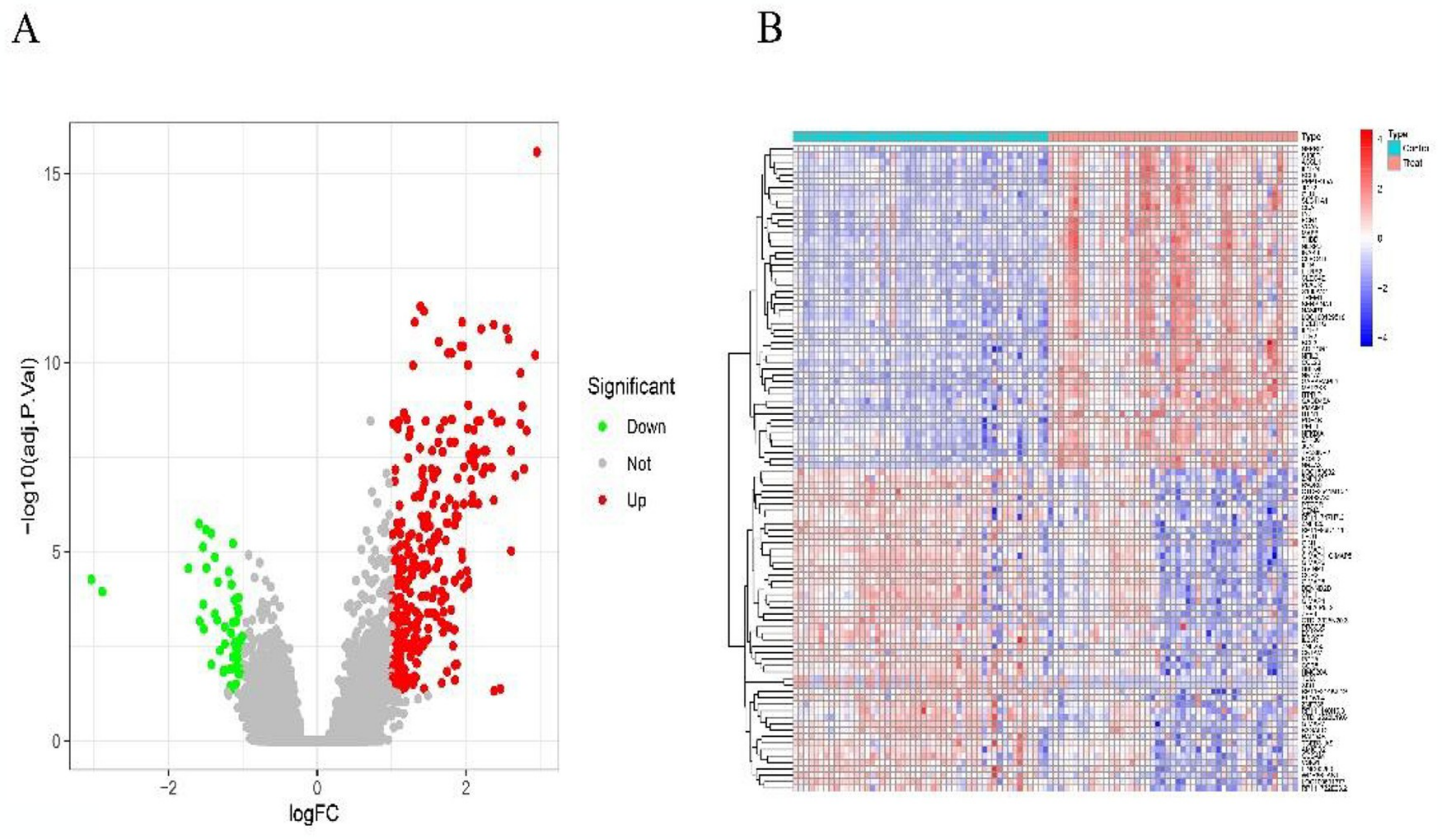
Differentially expressed genes in different types of AMI. (A)Volcano plot for differential expression analysis of GSE66360. (B)Heat map of GSE66360 differential expression analysis.

We downloaded the AMI dataset (GSE66360) from the GEO database and conducted differential expression analysis to identify the DEGs in the AMI dataset. Comparing the control group, we identified 340 DEGs in the AMI group, 285 up-regulated and 55 down-regulated (Fig 1A and 1B; S1 Table).

### 3.2 Construction of WGCNA network

**Fig 2:**
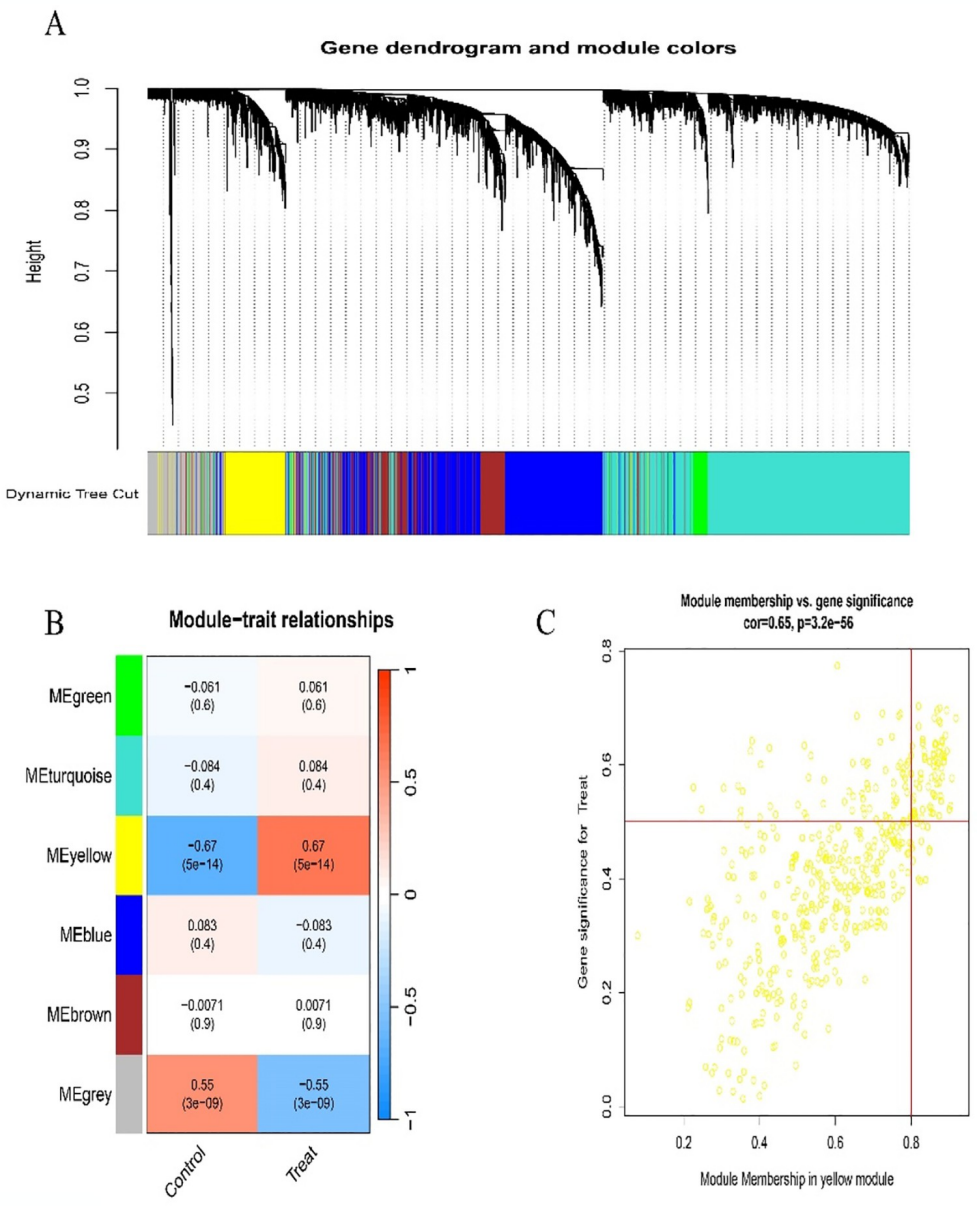
WGCNA and significant module recognition. (A)Dendrogram of all genes in the GSE66360 dataset clustered based on the topological overlap matrix(1-TOM). (B)Module-trait heat map of the correlation between clustered gene modules and AMI in the GSE66360 dataset. Each module contains the corresponding correlation coefficient and p-value. (C)In the GSE66360 data set, the yellow scatter plot of the module has the most significant positive correlation with AMI.

We performed WGCNA analysis on hub genes from the dataset (GSE66360) to assess the gene expression associated with AMI (Fig 2A). We identified six distinct modules (Fig 2B). Following a positive correlation coefficient analysis, we pinpointed the most significant correlation between the yellow module and AMI (Fig 2C; S2 Table).

### 3.3 Enrichment analysis

**Fig 3:**
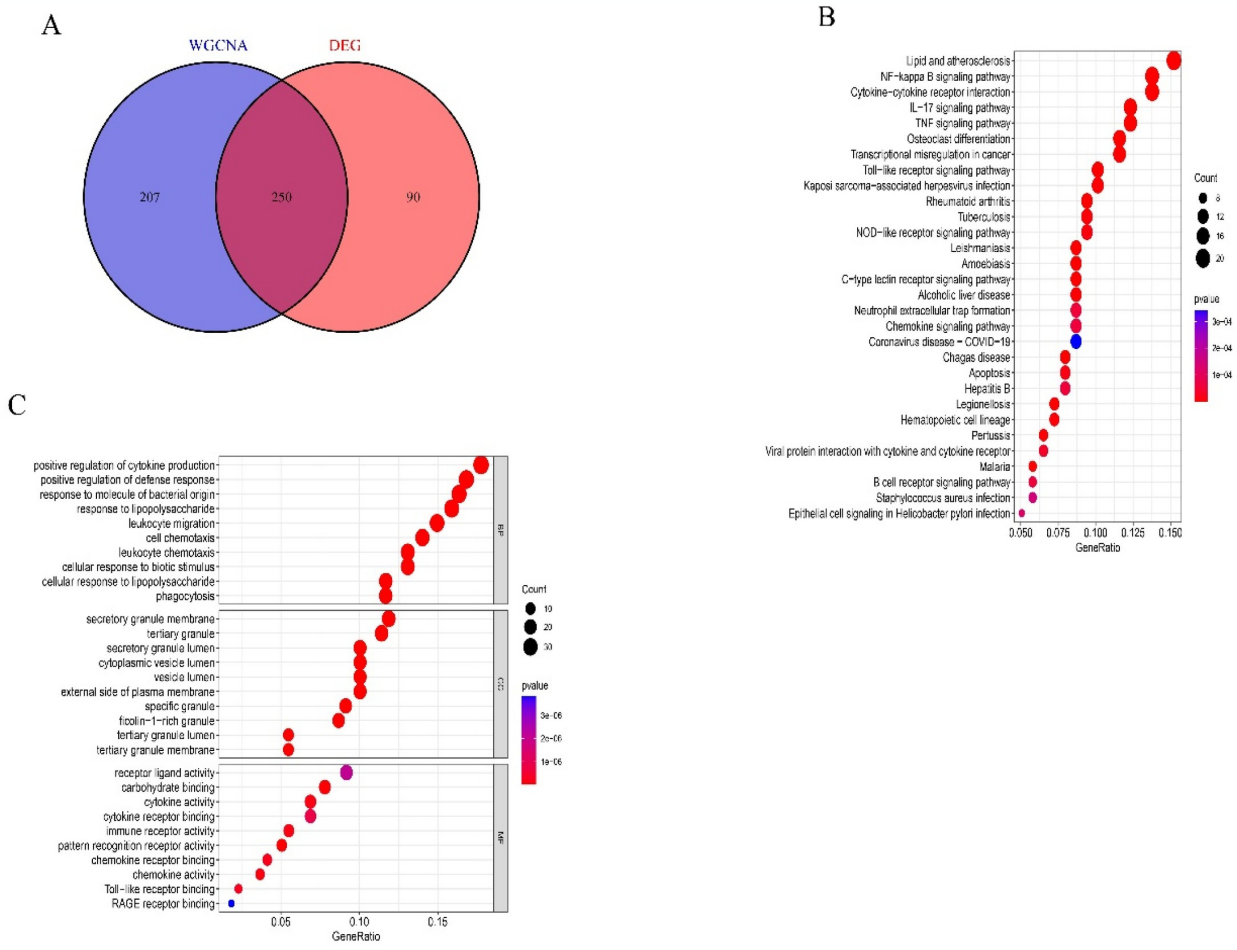
Screening and validation of candidate hub genes. (A)Venn diagram showing 250 overlapping intersection genes. (B)Pathway analysis of KEGG candidate hub genes. (C) GO enrichment analysis of candidate hub genes.

We utilized the WGCNA method to identify intersecting genes between disease-related hub genes and DEGs. Ultimately, we identified 250 intersection genes, which were designated as candidate hub genes, potentially crucial in the onset and progression of AMI (Fig 3A). Subsequently, we conducted GO and KEGG enrichment analyses to investigate the potential biological functions of the intersecting genes (Fig 3B and 3C). GO analysis demonstrated that the intersecting genes are significantly associated with positive regulation of cytokine production, positive regulation of defense response, secretory granule membrane, tertiary granule, carbohydrate binding, cell chemotaxis, and immune receptor activity. Additionally, KEGG analysis demonstrated that candidate hub genes predominantly influence lipid metabolism and atherosclerosis, NF—kappa B signaling pathway, cytokine—cytokine receptor interaction, IL−17 signaling pathway, and TNF signaling pathway.

### 3.4 PPI network analysis construction and hub gene screening

**Fig 4:**
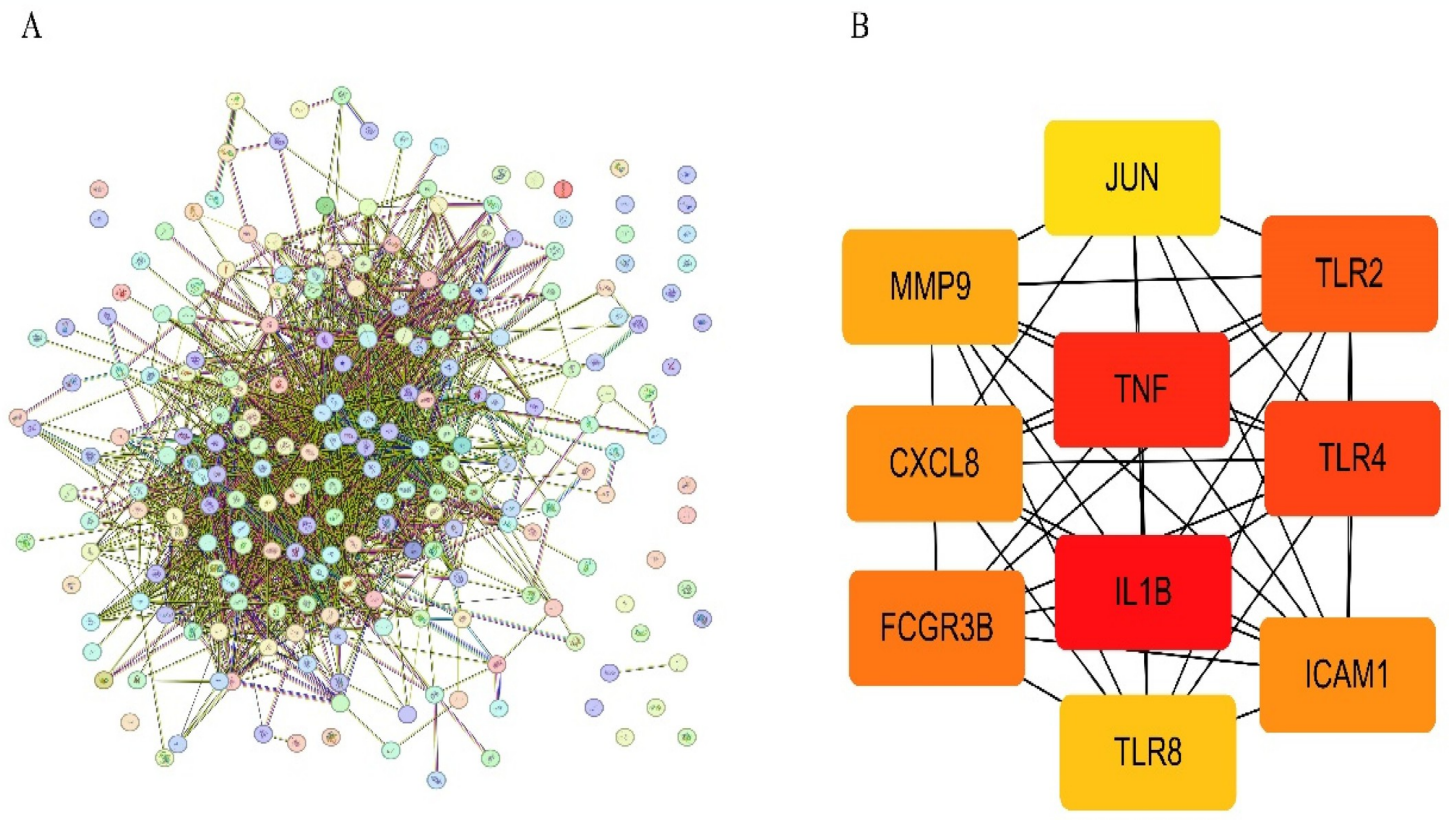
PPI network. (A)PPI network of intersection hub genes. (B)Protein interaction network core gene map (the darker the color, the higher the score).

We used the STRING online tool to establish a PPI network for the intersecting genes (Fig 4A). We visualized the top ten genes within the connected nodes using the CytoHubba plugin of Cytoscape software (Fig 4B). The 10 hub genes include IL1B, TNF, TLR4, TLR2, FCGR3B, MMP9, CXCL8, TLR8, ICAM1, and JUK.

### 3.5 Nomogram construction

**Fig 5:**
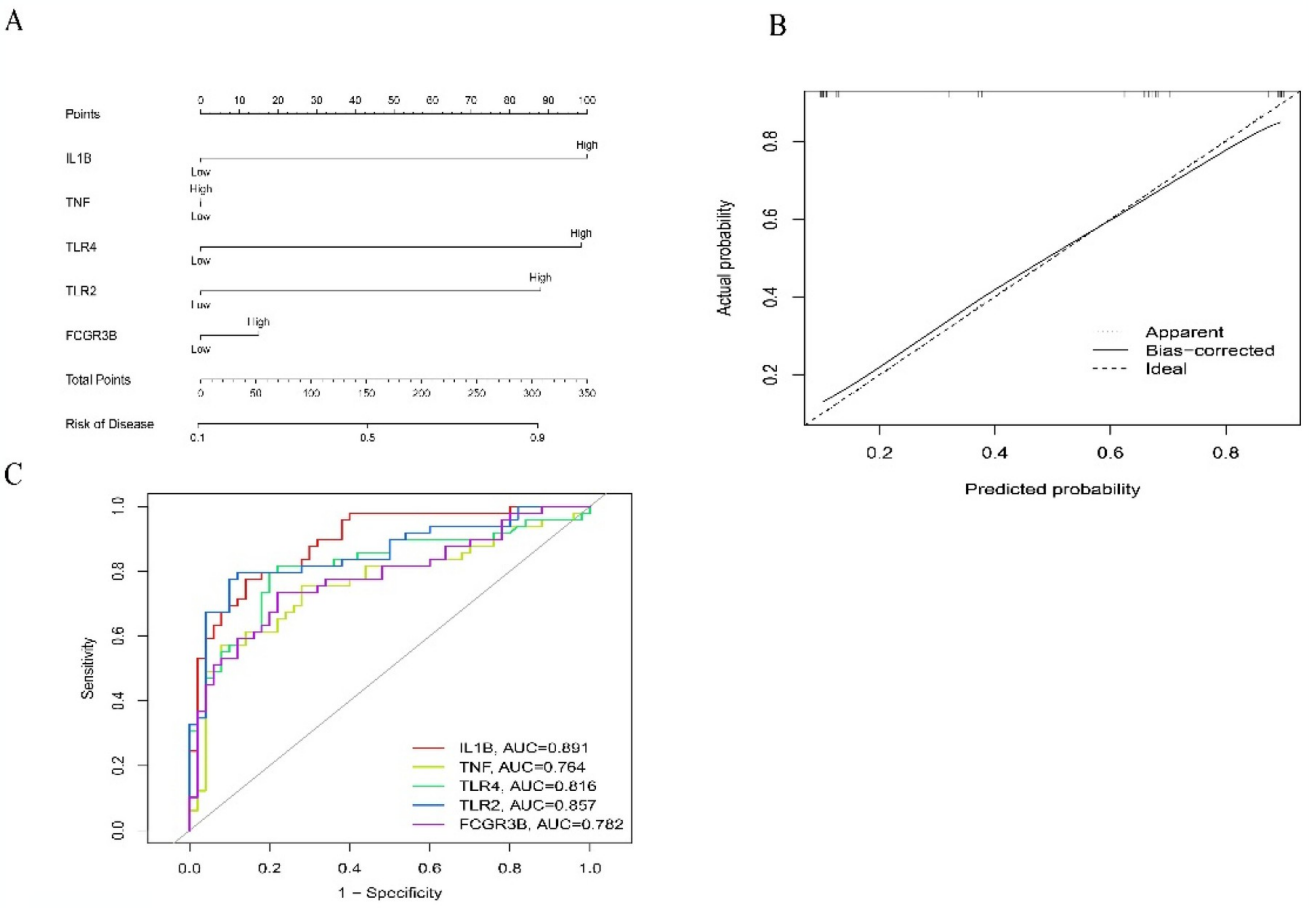
Nomogram predicting AMI risk. (A)Hub gene nomogram model. (B)Calibration curve assessing the predictive ability of the nomogram. (C) ROC curve to evaluate the diagnostic efficacy of the nomogram model and each hub gene.

A nomogram model was created to forecast the risk of AMI (Fig 5A). By calibration curves, we assessed the predictive performance of the nomogram, demonstrating its high accuracy in forecasting the risk of AMI (Fig 5B). Then, we established the ROC curves of 5 hub genes (IL1B, TNF, TLR4, TLR2, and FCGR3B) to assess their diagnostic effectiveness. The AUC of the ROC curve≥0.7 is considered clinically valuable (Fig 5C). The calculated AUC values for IL1B, TNF, TLR4, TLR2, and FCGR3B were 0.891, 0.764, 0.816, 0.857, and 0.782, respectively (Fig 5C).

### 3.6 Immune cell infiltration

**Fig 6:**
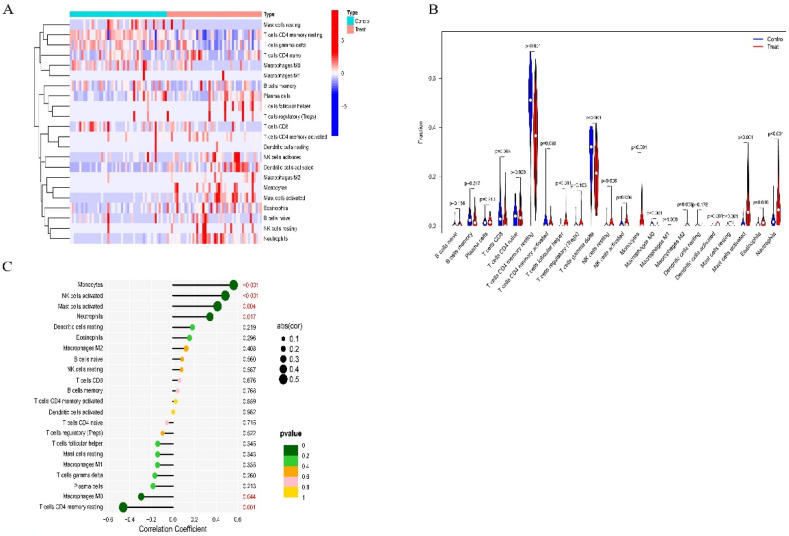
Immune relevance of TNF in AMI. (A) Relative distribution of 22 immune cell types in all AMI samples. (B) Difference in immune cell infiltration between AMI and controls. (C)Correlation between TNF and infiltration immune cells in AMI.

We used the CIBERSORT algorithm to analyze immune cell infiltration proportions between the AMI and control groups (Fig 6A). The results revealed significant differences between the groups. The AMI group exhibited higher proportions of T cells follicular helper, Monocytes, Macrophages M2, Dendritic cells activated, NK cells activated, Mast cells, activated Neutrophils, Eosinophils, NK cells resting. However, the infiltration proportions of T cells CD4 memory resting, T cells gamma delta, Macrophages M0 and Mast cells resting were lower in the AMI group (Fig 6B). According to the correlations with 22 types of immune cells, TNF showed positive correlations with Monocytes (r = 0.56), NK cells activated (r = 0.48), Mast cells activated (r = 0.41), and Neutrophils (r = 0.34). Conversely, TNF exhibited negative correlations with T cells CD4 memory resting (r = −0.46) and Macrophages M0 (r = −0.29) (Fig 6C). Hence, it can be inferred that immune cell infiltration is related to gene expression differences.

### 3.7 There is a causal relationship between TNF and the risk of myocardial infarction

**Fig 7:**
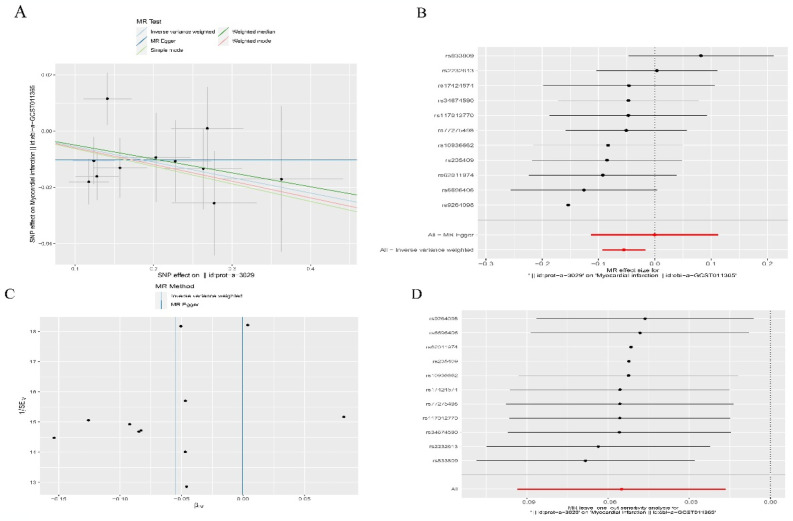
Results of mendelian randomization studies. (A)Scatter plot showing the causal effect of TNF on AMI risk. (B)Forest plot showing the causal effect of each SNP on AMI risk. (C) Funnel plot showing overall heterogeneity in MR estimates of the effect of TNF on AMI. (D)Leave-one-out plot visualizing the causal effect of’ TNF on AMI risk when one SNP is omitted.

The characteristics of SNPs related to TNF and AMI are presented in S3 Table. There are no weak instrumental variables in the SNPs. Visualization of causal effects between related genetic variables and AMI (Fig 7A and 7B). We assessed the causal relationship between TNF and AMI. Using the IVW, we identified a significant association between TNF and the risk of AMI, with an OR of 0.946 (95% CI 0.911−0.984, p = 0.005). The MR Egger test evaluation showed that there was no heterogeneity in the study (p = 0.340) (Fig 7C). The MR Egger regression intercept test, assessing data pleiotropy, demonstrated no pleiotropy in the data (p = 0.340), further confirming the robustness of the results. In the leave-one-out sensitivity test, no polymorphisms were identified, and no individual SNPs significantly influenced the results, affirming the reliability of the causal effect estimates (Fig 7D).

## 4. Discussion

AMI stands as a major global contributor to cardiovascular disease-related deaths and disabilities, yet its precise pathological mechanisms remain unclear [2, 13]. The occurrence of myocardial infarction is usually a sudden symptom, which not only has serious clinical manifestations but is often accompanied by complex complications, seriously affecting the patient’s survival and prognosis. Even though current evidence-based treatments have been well developed, AMI remains the main cause of chronic heart failure [14, 15]. Especially for patients with large-area infarction complicated by cardiogenic shock, their treatment remains particularly challenging. Timely and effective intervention is crucial in reducing AMI mortality rates and complications [16]. Modern molecular biology has created opportunities to study the involvement of gene expression related to AMI in the initiation and advancement of AMI. This progress offers promising prospects for improving clinical outcomes for patients [17–19]. Through the quantification of gene expression levels in biological samples, we can obtain comprehensive genome-wide expression data, and then perform genome-wide association analysis to find better targets for the treatment of AMI.

Early diagnosis of AMI is crucial, prompting researchers to actively analyze and verify AMI-related biomarkers to identify optimal treatment targets. When considering AMI as a therapeutic target, it is essential to stress that the pathology is not only dynamic but also highly individualized. Therapeutic interventions must therefore be adaptable to changes in the patient’s condition and the stage of AMI. Discussing the variability in timing and how it could affect study results is important for developing flexible, responsive treatment protocols. This consideration ensures that interventions remain effective across different stages of AMI and can be tailored to individual patient needs based on the timing and progression of the disease [20–22]. In a recent study, PPI analysis was employed to identify six hub genes associated with AMI, TLR2, IL1B, LILRB2, FCER1G, FPR1, and MMP9 [23]. Previous research identified Cxcl1, Cxcl2, Cxcl5, and Mmp8 as diagnostic biomarkers for AMI [24]. Additionally, studies have indicated elevated levels of ADM gene expression in cardiac tissue and overexpression of genes KLRD1 and LILRB3 in the peripheral blood of AMI patients, accelerating cardiomyocyte apoptosis [25, 26]. During the occurrence of AMI, the FOSL2 gene in the peripheral blood of patients exhibited low expression, indicating its protective effect on cardiomyocytes [26]. The gene CAMP activates the Akt and ERK pathways, inducing FoxO3a phosphorylation and subsequent nuclear export, thereby reducing cardiomyocyte apoptosis in AMI [27]. In another study, six genes—GIMAP7, GIMAP4, CCR2, NR4A2, CSTA, and S100A12—were identified as hub genes of AMI, with NR4A2 being the most valuable gene for MI diagnosis and demonstrating a protective effect on cardiomyocytes [28]. However, these studies lacked verification and evaluation of the accuracy and effectiveness of candidate biological diagnostic markers through clinical trials. When studying related diseases, it is essential to employ comprehensive bioinformatics methods. In this study, WGCNA analysis was utilized to screen the hub genes of AMI. Ten genes, including IL1B, TNF, TLR4, TLR2, FCGR3B, MMP9, CXCL8, TLR8, ICAM1, and JUK, were identified as candidate hub genes for AMI. The predictive ability of the nomogram was evaluated using a calibration curve, demonstrating its specificity and sensitivity in predicting the risk of AMI. AUC values for five central genes (IL1B, TNF, TLR4, TLR2, and FCGR3B) were calculated, and an ROC curve was plotted to assess diagnostic efficacy.

Increasingly, microarray datasets are utilized for predicting disease biomarkers and therapeutic targets. We obtained the GSE66360 data set and conducted bioinformatics analysis, identifying 340 DEGs in the AMI group, 285 up-regulated and 55 down-regulated. Some cytokines (IL-10, IL-5, VEGF, and TGF-β) participate in the positive regulation process of AMI through autocrine or paracrine means [29, 30]. These results underscore the importance of cytokine regulation in AMI and suggest they may be hub genes influencing AMI’s occurrence and progression. Inflammation and oxidative stress play pivotal roles in AMI [31]. The NF-κB signaling pathway is involved in the inflammatory response. It has been reported through studies that adiponectin inhibits the inflammatory response of aortic atherosclerotic plaques by suppressing the activation of the NF-kB signaling pathway and the expression of pro-inflammatory genes [32]. The IL-17 signaling pathway induces the expression of pro-inflammatory transcription factors by activating NF-kB, MAPK, and C/EBP cascade reactions, participating in AMI’s inflammatory response [33]. The KMT2B gene promotes the high expression of pro-inflammatory genes and induces cardiomyocyte damage and apoptosis through the RFK-mediated TNF-α/NOX 2 signaling pathway [34]. However, whether cytokines and pathways such as JNK-NF-kappa B, IL-17, or TNF are interrelated in AMI requires further investigation.

Cytokines, single-gene products, serve as crucial mediators in inflammation and immune responses [35]. They play diverse roles in cardiovascular diseases, primarily by binding to cell membrane receptors, and initiating specific intracellular signaling pathways, thereby activating transcription factors and regulating cell functions. Stimulatory and inhibitory cytokines have increasingly become promising therapeutic targets in the cardiovascular field [36]. FCGR3B is a key mediator in the inflammatory response. It activates neutrophils by binding to immune complexes, and activated neutrophils can cause ischemic damage to tissues [37, 38]. Our research indicates that FCGR3B is one of the critical hub genes in AMI. Therefore, inhibiting FCGR3B could offer a new therapeutic approach for AMI. TLRs (Toll-like receptors) act as major receptors for innate and adaptive immunity, playing a pivotal role in non-infectious pathological cardiovascular conditions such as myocardial infarction and heart failure [39]. TLR4 and TLR2 activation following myocardial ischemic injury triggers the NF-kappa B pathway and subsequent expression of pro-inflammatory genes, inducing an inflammatory cascade, increasing myocardial infarction size, and impacting cardiac remodeling [40]. Our study once again proved that TLR4 and TLR2 are one of the key genes of AMI. Consequently, inhibiting these receptors may offer an effective pathway for the treatment of AMI. Interleukin-1 beta(IL-1β)is a pro-inflammatory cytokine that plays a crucial role in inflammation and immune responses. IL-1β actively participates in the immune response, the generation of inflammatory mediators, and the ventricular remodeling process after AMI by driving caspase-3 activity [41]. These findings further support our research, highlighting IL1B as one of the central genes in this context. Our study further identifies potential therapeutic targets for AMI.

According to the analysis conducted using Cytoscape software, TNF is the most significant hub gene in the PPI network. TNF is primarily produced by the heart, with both cardiac macrophages and cardiomyocytes being responsible for its production [42]. The TNF superfamily (TNFSF) has 19 ligands and 29 receptors, serving as crucial mediators and regulators in human immune and inflammatory responses [43]. Mounting evidence demonstrates that TNF is a potent pro-inflammatory cytokine implicated in the pathogenesis of various cardiovascular diseases [42, 44, 45]. TNF-α, for instance, stimulates the myocardium to release free radicals, leading to cardiomyocyte dysfunction after myocardial ischemia. Myocardial ischemia-reperfusion (I/R) activates macrophages to produce TNF-α, inducing the overexpression of intracellular adhesion molecule-1 (ICAM-1) on cardiomyocytes. This process promotes the migration of neutrophils and neutrophil accumulation in the reperfused myocardium. The adhesive interactions between cardiomyocytes ultimately lead to the release of inflammatory mediators, such as oxygen free radicals, leukotrienes, and cytokines, thereby contributing to myocardial ischemia and affecting myocardial function [46]. Ischemia and hypoxia have been shown to stimulate and induce the secretion of large amounts of TNF-α by cardiomyocytes and cardiac macrophages. The interaction between TNF-α and TNFR1 primarily contributes to the inflammatory response and ventricular remodeling in acute myocardial infarction (AMI), leading to cardiomyocyte apoptosis and cardiotoxicity [47]. Xu et al. discovered that estrogen can inhibit TNF-α levels and reduce the cardiac expression of TNF-α receptors. This reduction in TNF-α activity helps in mitigating cardiomyocyte damage, apoptosis, and improving overall cardiomyocyte function during the inflammatory response of AMI [48]. However, there is evidence suggesting that TNF plays a dual role in the occurrence and development of AMI, with physiological levels showing a protective effect on the heart, as physiological levels of TNF exert a protective effect on the heart [49]. Activated TNF is a key mediator in promoting cell survival during ischemic preconditioning and postconditioning. Low doses of TNF can rapidly activate the transcription factor NF-kB, which in turn mediates the cardioprotective effects of various forms of ischemia and drug preconditioning. This activation helps in reducing damage caused by ischemia-reperfusion and also regulates the functioning of cardiomyocytes [50]. Lecour et al. discovered that in the AMI mouse model, an optimal dose of TNF can effectively decrease the size of myocardial infarction and aid in the restoration of myocardial function post-infarction [51]. TNF has been implicated in the regulation of troponin T, a protein found in cardiac muscle cells that is released into the bloodstream following myocardial injury. Several studies have investigated the relationship between TNF and troponin T levels in various cardiac conditions. TNF has been shown to upregulate the expression of troponin T in cardiac muscle cells, leading to increased levels of troponin T in the bloodstream. This upregulation occurs as a result of TNF-induced myocardial injury and inflammation, which can occur in conditions such as myocarditis, myocardial infarction, and heart failure [52].Additionally, elevated levels of TNF have been associated with increased troponin T release and worse clinical outcomes in patients with acute coronary syndromes and heart failure. Our study confirmed that TNF has a protective effect on cardiomyocytes during inflammation and oxidative stress in AMI. Therefore, TNF can serve as a potential biomarker for treating AMI.

This study conducted the first analysis of the causal effect of serum TNF levels on the risk of AMI using a two-sample MR approach based on GWAS data. The results suggest that there may be a causal effect of serum TNF levels in reducing the risk of AMI. MR circumvents the systematic biases influencing the outcomes of observational studies, such as confounding factors and reverse causal effects, enhancing the reliability of the results. To mitigate the influence of confounding factors, we exclusively included participants from the American population in our analysis. Additionally, we conducted MR-Egger regression tests and leave-one-out sensitivity analyses, which indicated no presence of horizontal pleiotropy or data sensitivity.

Our study has several limitations. Firstly, we were constrained to a single dataset due to the limited availability of AMI datasets in the GEO database, potentially affecting result accuracy due to the small sample size. Secondly, our analysis focused exclusively on hub genes and their potential roles in relation in AMI through bioinformatics approaches. To validate and expand our findings, it will be necessary to conduct in-depth in vivo and in vitro experiments in the future.

## 5. Conclusions

We established a co-expression network using WGCNA and identified hub genes associated with AMI. Comprehensive bioinformatics analysis showed that low-dose TNF can inhibit inflammatory and oxidative stress responses in AMI, exerting a protective role in the heart. This discovery holds potential for enhancing early diagnosis and effective treatment strategies for AMI. Furthermore, it provides a certain reference value for further exploring the role of these biomarkers in AMI diagnosis and treatment.

## Supporting information

S1 Table Differential gene expression. (XLS)

S2 Table WGCNA related module gene expression. (XLS)

S3 Table The characteristics between exposure factors and outcomes. (CSV)
